# Ergot

**Published:** 1841-11

**Authors:** 


*Ergot.*—Mr. Wright inclines to the opinion which ascribes the formation of the excrescence to the combined influence of atmospheric warmth, and moisture. He is the more inclined to this belief, from the circumstance of the ergot being much more common in the district of Sologne than elsewhere, this district being possessed of those properties which favor such a conclusion, viz. moist rich soil, atmospheric warmth, and sheltered situation. Grains also occur only half of which are ergotted, the other half being healthy; an insuperable objection to the opinion, that ergot is produced by the growth of a fungus, which would equally attack all parts of the grain. Besides, from his own experiments, he found that ergot in powder or substance, sowed with rye, failed to produce the disease on the growing plant, nor did it even succeed, when he watered freely and daily, the growing plants with water, in which ergot had been steeped. The application of the powder of ergot to the growing ears of rye, likewise failed to produce ergotted grains. The excrescences remarked at the upper extremity of the grains he regarded as the stigmata altered by disease. Mr. Wright's conclusions were further confirmed by the fact of his discovery, that ergot contained a considerable quantity of fecula, 26 per cent., a substance which could not have been expected, if the disease were produced by a fungus, and which Mr. Wright has the merit of discovering, no previous analyist of the substance making mention of it.—*Edinburgh Med. and Surg. Jour., Jan.* 1841; *Amer. Jour. Med. Sci.*

